# Extracellular vesicles: an emerging player in retinal homeostasis

**DOI:** 10.3389/fcell.2023.1059141

**Published:** 2023-04-25

**Authors:** Amit Chatterjee, Ruchira Singh

**Affiliations:** ^1^ Department of Ophthalmology, University of Rochester, Rochester, NY, United States; ^2^ Department of Biomedical Genetics, University of Rochester, Rochester, NY, United States; ^3^ Center for Visual Science, University of Rochester, Rochester, NY, United States; ^4^ UR Stem Cell and Regenerative Medicine Center, University of Rochester, Rochester, NY, United States

**Keywords:** extracellular vesicles, microvesicles, retina, retinal degeneration, exosomes

## Abstract

Extracellular vesicles (EVs) encompass secreted membrane vesicles of varied sizes, including exosomes (−30–200 nm) and microvesicles (MVs) that are ∼100–1,000 nm in size. EVs play an important role in autocrine, paracrine, and endocrine signaling and are implicated in myriad human disorders including prominent retinal degenerative diseases, like age related macular degeneration (AMD) and diabetic retinopathy (DR). Studies of EVs *in vitro* using transformed cell lines, primary cultures, and more recently, induced pluripotent stem cell derived retinal cell type(s) (e.g., retinal pigment epithelium) have provided insights into the composition and function of EVs in the retina. Furthermore, consistent with a causal role of EVs in retinal degenerative diseases, altering EV composition has promoted pro-retinopathy cellular and molecular events in both *in vitro* and *in vivo* models. In this review, we summarize the current understanding of the role of EVs in retinal (patho)physiology. Specifically, we will focus on disease-associated EV alterations in specific retinal diseases. Furthermore, we discuss the potential utility of EVs in diagnostic and therapeutic strategies for targeting retinal diseases.

## 1 Introduction

### 1.1 EVs: origin, nomenclature, and characteristics

Vesicles are membrane-enclosed lumens of varying size from 100 nm to 1,000 nm (Colombo et al., 2014). Vesicles can reside and function within the cytoplasm and/or be secretory. It is well-established that cytoplasmic vesicles such as endosomes, lysosomes, and peroxisomes play an important role in protein trafficking and localization (Sanderfoot and Raikhel 1999). In contrast, our knowledge of secretory vesicles or extracellular vesicles (EVs) in cellular homeostasis and cell-cell communication is still rudimentary. This is despite the fact that EV secretion is evolutionarily conserved from prokaryotes to eukaryotic cells and has been documented to occur in most human cell types. In fact, due to the high heterogeneity of the secreted vesicles, the nomenclature for characterizing EVs is still being developed (Théry et al., 2006; Théry et al., 2018). Broadly, based on the cellular source of origin, EVs comprise of three categories: exosomes (also known as small extracellular vesicles or sEVs), microvesicles (MVs), and apoptotic bodies (*summarized* in Table 1). Furthermore, physical traits and biochemical composition have been utilized extensively to characterize EVs (*summarized* in Table 1). (Théry et al., 2006; Colombo et al., 2014; Kowal et al., 2016; Costa Verdera et al., 2017; Skotland et al., 2017; Todorova et al., 2017; Chaudhuri et al., 2018; Théry et al., 2018; Catalano and O'Driscoll 2020; Joshi et al., 2020; Ye et al., 2021)

**TABLE 1:** Summary of EV categories with description of EV properties including physical traits and biochemical composition based on published studies.

Type of EVs properties	Apoptotic bodies	Microvesicles	Exosomes	References
***Source of origin***	Apoptotic cells	Outward budding of the plasma membrane	Endosomes, Intraluminal vesicles and Multivesicular body	*(* *Colombo et al. 2014* *)*
***Biogenesis Pathways***	Apoptotic pathway	Stimulus dependent and calcium dependent	ESCRT dependent, tetraspanin dependent, stimulus dependent and ceramide dependent	*(* *Colombo et al. 2014* *;* *Todorova et al. 2017* *;* *Théry et al. 2018* *)*
***Size***	50 nm–5000 nm	100 nm–1000 nm	30 nm–150 nm	*(* *Colombo et al. 2014* *;* *Todorova et al. 2017* *;* *Théry et al. 2018* *)*
***Morphology***	Vesicle like structure and heterogeneous morphology	Vesicle like structure and heterogeneous morphology	Cup shape morphology	*(* *Colombo et al. 2014* *;* *Todorova et al. 2017* *;* *Théry et al. 2018* *)*
***Sedimentation***	5,000–16,000 X g	16,000–25,000 X g	100,000–160,000 X g	*(* *Théry et al. 2006* *;* *Colombo et al. 2014* *;* *Théry et al. 2018* *)*
***Uptake by recipient cells***	NA	Fusion, Endocytosis, Ligand binding	Fusion, Endocytosis, Ligand binding	*(* *Tian et al. 2014* *;* *Costa Verdera et al. 2017* *;* *Joshi et al. 2020* *)*
***Pharmacological**; **Inhibitors***	Caspase inhibitors	Calpeptin, Y27632, pantetheine, imipramine, GW4869, manumycin A, bisindolylmaleimide I, U0126, clopidogrel, imatinib, NSC23766, chloramidine, cytochalasin D and sulfisoxazole	Calpeptin, Y27632, pantetheine, imipramine, GW4869, manumycin A, bisindolylmaleimide I, U0126, clopidogrel, Imatinib, NSC23766, Chloramidine, cytochalasin D and sulfisoxazole	*(* *Catalano and O'Driscoll 2020* *)*
***Cellular composition***	Cell organelles present	Cell organelles present	Not present	*(* *Colombo et al. 2014* *;* *Todorova et al. 2017* *;* *Théry et al. 2018* *)*
***DNA***	Present	Present	Present	*(* *Théry et al. 2006* *)*
***RNA***	Ribosomal RNA	mRNA	Enriched in small non-coding RNAs such as miRNA, Piwi RNA, circular RNA and long non-coding RNA	*(* *Chaudhuri et al. 2018* *;* *Théry et al. 2018* *;* *Ye et al. 2021* *)*
***Proteins***	Apoptotic proteins and histone proteins	Ribosomal proteins, mitochondrial proteins and cytokines	ECM proteins, endosomal proteins, proteasomal proteins, angiogenic proteins	*(* *Théry et al. 2006* *;* *Kowal et al. 2016* *;* *Théry et al. 2018* *)*
***Enriched protein marker***	Caspase3 and histones	Selectins, integrins, and CD63	CD81, CD63, CD9, Alix, Syntenin-1	*(* *Théry et al. 2006* *;* *Colombo et al. 2014* *;* *Théry et al. 2018* *)*
***Lipids***	NA	Enriched in sphingomyelinase and phosphatidylserine	Enriched in sphingolipid, cholesterol, and phospholipids	*(* *Skotland et al. 2017* *)*
***Protein to Lipid ratio***	High	Medium	Low	*(* *Théry et al. 2006* *;* *Colombo et al. 2014* *;* *Skotland et al. 2017* *;* *Théry et al. 2018* *)*

### 1.2 Overview of EVs in normal and pathological conditions

The major role of EVs in normal physiology is intercellular communication and removal of molecular waste material as a means for cell maintenance (Yáñez-Mó et al., 2015). Note that the lipid membrane of the EVs is critical in protecting the molecular constituents of the EVs from degradation by the enzymes in the surrounding fluids (Yáñez-Mó et al., 2015). Importantly, protection of the EV molecular cargo in bodily fluids permits their function in endocrine signaling between the parental cell and distant cell type(s) (Turturici et al., 2014; Borgovan et al., 2019).

Uptake of EVs by a particular recipient cell has been shown to depend upon five factors: 1) the donor cell type (Del Fattore et al., 2015; Sancho-Albero et al., 2019; Hamzah et al., 2021), 2) the composition of the EVs (Mulcahy et al., 2014; Zhang Y et al., 2019), 3) the recipient cell type (Horibe et al., 2018), 4) the donor and recipient cell’s physiological state (Parolini et al., 2009; Sung et al., 2020), and 5) a mutual recognition of ligands or receptors between the recipient cell and EVs (Viñas et al., 2018; Joshi et al., 2020). For instance, monocyte derived EVs are selectively taken up by platelets expressing P selectins (SELP) (Oggero et al., 2019). Although the exact mechanism(s) of EV uptake by a specific cell type is controversial, uptake of EVs by the recipient cells can occur through different mode(s) of endocytosis, including clathrin-dependent or -independent endocytosis, caveolin-mediated, macropinocytosis, phagocytosis, and lipid raft-mediated endocytosis (Tian et al., 2014; Costa Verdera et al., 2017; Joshi et al., 2020; Kwok et al., 2021).

The cellular source of the EV origin has also been shown to influence the impact of EVs on the recipient cell’s physiology (Patel et al., 2016; Cosenza et al., 2018; Gonzalez-Nolasco et al., 2018; Ranjit et al., 2018; Wang et al., 2020; Wei et al., 2020; Maldonado et al., 2021; Shahin et al., 2021; Shen et al., 2021). For example, EVs from adipose derived stem cells can alleviate oxidative stress and inflammation in macrophages (Shen et al., 2021). In contrast, MVs and exosomes from amnion epithelial cells under oxidative stress show a distinct proinflammatory cytokine profile and cause synergistic inflammation in uterine cells leading to the initiation of parturition (Shahin et al., 2021). It is also noteworthy that EVs from diseased cells can influence the characteristics of normal, unaffected cells. For example, in tuberous sclerosis complex (TSC) genetic disorder, EVs from *TSC-1* null cells transform the phenotype of neighboring wild-type cells to mimic the diseased cell (Patel et al., 2016). Similarly, human monocytes exposed to alcohol release exosomes, which polarize neighboring monocytes to M2 macrophages and consequently regulate inflammation (Saha et al., 2016).

Overall, given that EVs can impact cell function and cell-cell communication through autocrine, paracrine, and endocrine signaling mechanisms, it is not surprising that EVs have been demonstrated to play a central role in tissue homeostasis and influence the development of several human disorders including cardiovascular diseases (Bellin et al., 2019; Stamatikos et al., 2020), cancer (Sharma et al., 2020; Chen et al., 2021; Müller Bark et al., 2021), infectious diseases (e.g., tuberculosis, hepatitis B virus infection, microbial keratitis, Toxoplasmosis, etc.) (Abrami et al., 2013; Cosset and Dreux 2014; Barclay et al., 2017; Zhang W et al., 2018), and neurodegenerative diseases (e.g., Alzheimer, Parkinson, stroke, etc.) (Sharples et al., 2008; Abner et al., 2016; Howitt and Hill 2016; Zagrean et al., 2018; Liu W et al., 2019).

From the perspective of the retina, intercellular communication between various retinal cell-types (e.g., neurons, glia, endothelial cells, pericytes, immune cells) and extracellular matrix (ECM) constituents is essential for maintaining the normal tissue function and physiology. Accordingly, several recent studies have focused on 1) identification and characterization of EVs in the retina (reviewed in Sections 2.1, 2.2), 2) pathogenic role of EVs in retinal degenerative diseases (Section 2.3), 3) utility of EVs as a biomarker for retinal diseases (Section 3), and 4) therapeutic applications of EVs in the retina (Section 4).

## 2 Molecular insights into EVs in the retina

### 2.1 EVs in the ocular biofluids

The characterization of EVs in the eye *in vivo* has primarily relied on biofluids. Specifically, EVs have been isolated from ocular biofluids, aqueous humor, tears, and the vitreous humor (VH), the proximal biofluid of the retina (Li et al., 2014; Welton et al., 2017; Wu and Liu 2018; Zhao et al., 2018; Han et al., 2021). Note that VH is considered a filtrate of serum and is the preferred biofluid for postmortem biochemical investigation of ocular pathologies because of its large volume and ease of accessibility (Thierauf et al., 2009). Compositionally VH primarily consists of water, collagen, and hyaluronic acid and cytoplasmic resident proteins such as ANXA1, CSNK1A1, GSK3B, ROCK1, and various mitogen-activated protein kinases and phosphatases as well as several secreted proteins, including complement proteins (e.g., C3 and CFH), FN1, MMP2, and TIMP1 (Murthy et al., 2014; Skeie et al., 2015; Zhao et al., 2018).

A contribution of retinal cells to the VH proteome is likely, given that anatomically, the VH directly opposes the retina. To corroborate this postulation, we utilized a previously described approach and compared a published human VH proteome (Skeie et al., 2015) with the human retina proteome (Velez et al., 2018). The human VH proteome (Skeie et al., 2015) showed 47% similarity in protein composition to the human retina proteome (Velez et al., 2018). Furthermore, the VH proteome (Skeie et al., 2015) had an even higher degree of proteome commonality (53.13%) when compared to that of the fovea macula proteome (Velez et al., 2018). This suggests that different areas of the retina and/or specific retinal cell type(s) contribute to the VH proteome. Furthermore, because exosomes have been reported to be the major constituent of the VH (Ragusa et al., 2015; Zhao et al., 2018), it is plausible that retinal cell-secreted exosomes are major contributors to the VH proteome. However, the specific contribution of individual retinal cell type(s) to the EV population within the VH needs to be further investigated.

Pathologically, EV composition within the VH is altered in individuals with idiopathic floaters and patients with specific retinal diseases like age-related macular degeneration (AMD) and diabetic retinopathy (DR) (Koss et al., 2014; Schori et al., 2018). In addition to changes in the composition, the altered abundance of EVs has also been reported in several pathologic conditions (Chahed et al., 2010; Tumahai et al., 2016) For example, elevated concentrations of MVs were present in the VH of proliferative DR (PDR) patients compared to control subjects that did not undergo previous vitreous surgery (Chahed et al., 2010). Further substantiating the pathogenic potential of EVs isolated from the VH of PDR patients, intravitreal delivery of MVs isolated from PDR patients led to increased endothelial cell proliferation and pathologic angiogenesis in mice (Chahed et al., 2010). Similarly, increased levels of several proinflammatory markers (e.g., IL6) were observed in MVs isolated from the VH of patients undergoing surgical repair of retinal detachment compared to the control group (Tumahai et al., 2016). Notably, in this study the authors postulated that nearby retinal cells led to increased concentration of MVs in the VH (Tumahai et al., 2016).

Apart from VH, EVs have also been reported in other ocular biofluids, specifically tear (Grigor’eva et al., 2016; Aqrawi et al., 2017; Inubushi et al., 2020; Liu H et al., 2022; Zhou et al., 2022) and aqueous humor (Liao et al., 2012; Dismuke et al., 2015; Lerner et al., 2017; Hsu et al., 2018; Chen et al., 2019; Lerner et al., 2020; Gao et al., 2021; Gao et al., 2021). Exosomes were reported to be present in high concentration in tears from healthy donors and the DNA present inside the exosomes was mapped to the human genome (Grigor’eva et al., 2016). Furthermore, analysis of EVs from tear fluid at a single particle level revealed its heterogeneous nature and a possible role of tear EVs in clot formation and pathogen invasion (Liu H et al., 2022). Highlighting the altered composition of aqueous humor in retinal diseases, a higher number of exosomes and MVs were present in the aqueous humor of patients with neovascular AMD, polypoidal choroidal vasculopathy, and central retinal vein occlusion (Hsiao et al., 2021).

Altogether, published studies clearly show the presence of EVs in ocular biofluids, aqueous humor, tear, and VH. Furthermore, EVs present in the ocular biofluids are altered in several retinal diseases like AMD and DR. Therefore, it is plausible that alterations in EV composition of ocular biofluids like VH potentially contribute to pathological alterations in specific retinal diseases. Conversely, EV characteristics (e.g., amount, composition) of ocular biofluid likely reflect health of the surrounding tissues, like retina, and thus may be a useful diagnostic or therapeutic biomarker in various ocular disorders (see Section 3).

### 2.2 EVs in in vitro retinal cell model(s)

Pluripotent stem cell-derived organoid/cells, primary cells, and transformed cell lines provide a platform to study retina-associated EVs with cell-type specificity. In fact, several studies have used these *in vitro* models to characterize EVs, particularly exosomes, secreted by specific retinal cells (Table 2). EVs have been isolated from several ocular cells including astrocytes, microglia, photoreceptors, and RPE (Table 2). (Sreekumar et al., 2010; Biasutto et al., 2013; Hajrasouliha et al., 2013; Kang et al., 2014; Knickelbein et al., 2016; Klingeborn et al., 2017; Grimaldi et al., 2019; Maisto et al., 2019; Xu et al., 2019; Fukushima et al., 2020; Kang et al., 2020; Ke et al., 2020; Morris et al., 2020; Zhang et al., 2020; Ahn et al., 2021; Flores-Bellver et al., 2021; Zhang and Wang 2021; Zhou et al., 2021; Ren et al., 2022).

**TABLE 2:** Published studies evaluating extracellular vesicle (EV) constituent and function in specific retinal cell type(s) utilizing *in vitro* models.

Retinal cell type(s)	Type of EVs highlighted	Major EV constituent highlighted	Biological process/phenotype regulated by EVs	Disease involved	Reference
Mouse retinal astrocytes	Exosomes	COL18A1, CXCL1, CCL3	Neovascularization	AMD	***(*** *Hajrasouliha et al. 2013* ***)***
Human adult RPE	Exosomes	CRYAB	Sub-RPE deposits and neuroprotection	AMD	*(* *Sreekumar et al. 2010* *)*
Human fetal RPE	Exosomes	miRNA-21	Paracrine signaling between RPE and microglia	AMD	*(* *Morris et al. 2020* *)*
RPE (ARPE-19)	Exosomes	miR-494-3p	Mitochondrial dysfunction	AMD	*(* *Ahn et al. 2021* *)*
RPE (ARPE-19)	Exosomes	Phosphoproteins such as MAPK14	Oxidative stress	AMD	*(* *Biasutto et al. 2013* *)*
RPE (ARPE-19)	Exosomes	APAF1, CASP9	Drusen, Autophagy	AMD	*(* *Ke et al. 2020* *)*
RPE (ARPE-19)	Exosomes	VEGF	Neovascularization	AMD	*(* *Maisto et al. 2019* *)*
RPE (ARPE-19)	Exosomes	TIMP1, IGFBP3, MMP9	Neovascularization	AMD	*(* *Fukushima et al. 2020* *)*
RPE (ARPE-19)	Exosomes	PIGF2, MCP1	Neovascularization	AMD	*(* *Hajrasouliha et al. 2013* *)*
RPE (ARPE-19)	Exosomes	IL6, IL8, TNF, TGFB1	Immune Response	AMD	*(* *Knickelbein et al. 2016* *)*
RPE (ARPE-19)	Exosomes	miRNAs	Epithelial-mesenchymal transition	Proliferative vitreoretinopathy	*(* *Zhang et al., 2020* *)*
RPE (ARPE-19)	Exosomes	CTSD, C3, C5, C9, KRT8, KRT14, ACTA2, MYO1	Autophagy lysosomal pathway	AMD	*(* *Kang et al. 2014* *)*
Retinal organoids	Exosomes	miRNAs	Retinal development	NA	*(* *Zhou et al. 2021* *)*
Porcine RPE	Exosomes	ECM associated proteins	Retinal homeostasis	NA	*(* *Klingeborn et al. 2017* *)*
iPSC-RPE	Exosomes	Drusen associated proteins	Drusen Deposits	AMD	*(* *Flores-Bellver et al. 2021* *)*
Photoreceptor	Exosomes	Thioredoxin, PRKAA2	Neovascularization	Diabetic retinopathy	*(* *Ren et al. 2022* *)*
Microglia	Microvesicles	mRNAs, IL1B, CCL2, CCL3, C3	Neuroprotection	Glioma	*(* *Grimaldi et al. 2019* *)*
Microglia	Exosomes	VEGF, TGFB1	Neovascularization	Oxygen induced retinopathy	*(* *Xu et al. 2019* *)*
Microglia	Exosomes	miR-24-3p	Cell survival	AMD	*(* *Xu et al. 2019* *)*

Due to the ease of culture and known role of RPE cells in several retinal degenerative diseases, including AMD, studies on EVs in the *in vitro* retina cell model(s) have thus far primarily utilized an immortalized RPE cell line (ARPE-19) and to some extent, primary and induced pluripotent stem cell (iPSC)-derived RPE (Table 1). Notably, the use of these different cell types, iPSC *versus* primary *versus* an immortalized cell line (ARPE-19) for studying RPE-secreted EVs, has raised important concerns about variability in EV population between iPSC-RPE, primary RPE, and ARPE-19 cells. In fact, highlighting this very discrepancy, comparative analysis of published ARPE-19 (Kang et al., 2014) and iPSC-RPE (Flores-Bellver et al., 2021) derived exosome proteomes showed only 30.45% overlap. Similarly, only 12 of the 28 apically and 16 of 72 basally enriched proteins in the EV proteome of primary porcine RPE cultures (Klingeborn et al., 2017; Flores-Bellver et al., 2021) were present in the published apical and basal EV proteome of iPSC-RPE cultures (Klingeborn et al., 2017; Flores-Bellver et al., 2021). Although these comparisons are limited to RPE cells, they illustrate that exosomes isolated from cell lines *versus* primary cells *versus* iPSC-derived target cells might not truly represent the exosome population released from the *in vivo* cellular counterpart. It is plausible that the isolation of EVs from *ex vivo* retina tissue explant, as previously described for the brain tissue (Levy 2017), will help validate specific retinal cell model(s) for use in *in vitro* studies of EVs.

### 2.3 Pathogenic role of EVs in retinal degenerative diseases

Retinal degeneration is the final consequence of many retinal diseases, which encompass both monogenic (e.g., Sorsby’s fundus dystrophy) and multifactorial diseases such as AMD, Leber congenital amaurosis, Stargardt’s disease, and retinitis pigmentosa. Although the valuation of altered exosome composition in promoting disease-associated pathophysiology in retinal degenerative diseases is still at a nascent stage of research, preliminary studies of specific retinopathies have already provided evidence for exosome-mediated cellular dysfunction in disease development and progression.

#### 2.3.1 AMD

AMD affects the photoreceptor-RPE-choroid complex in the eye. Furthermore, AMD can cause two distinct clinical phenotypes, non-exudative (dry-type) and exudative (wet-type). Dry AMD is characterized by drusen, accumulation of extracellular lipid-protein rich deposits beneath the RPE monolayer, ECM thickening, and atrophy of RPE. In contrast, wet-AMD involves aberrant neovascularization (choroidal neovascularization; CNV) leading to vision loss.

Given AMD’s multi-tissue pathology affecting the comprehensive photoreceptor-RPE-choroid complex, EVs could also contribute to AMD pathophysiology by altering intercellular communication between the various cell type(s). For example, paracrine signaling by EVs could play an important role in promoting both RPE-mediated photoreceptor (apical) and choroidal (basal) dysfunction in AMD. Consistently, porcine RPE-secreted apical *versus* basal exosomes were shown to have distinct proteomic composition (Klingeborn et al., 2017). Specifically, although the number of apically *versus* basally secreted EVs by porcine RPE were similar, apically released EVs contained approximately twice the number of proteins compared to basally released EVs (Klingeborn et al., 2017). Consistent with a role of polarized EV secretion by RPE cells in retinal homeostasis, αB crystallin, a small heat shock protein known to have anti-apoptotic and anti-inflammatory function is present in apically-secreted RPE exosomes, and likely provides neuroprotection to the neural retina/photoreceptors during oxidative stress (Sreekumar et al., 2010; Bhat and Gangalum 2011). In fact, consistent with a highly-controlled directional mechanism of EV secretion being differentially regulated in AMD, cigarette smoke exposure, a prominent modifiable risk factor for AMD, led to increased secretion of apically released EVs in iPSC-RPE cultures (Flores-Bellver et al., 2021). Conversely, exosomes isolated from a photoreceptor cell line (661W) were shown to regulate autophagy and phagocytosis by RPE cells (Ren et al., 2022). From the perspective of paracrine signaling, RPE exosome-mediated miRNA transfer has also been shown to contribute to the regulation of microglia function in the aging retina by inducing a proinflammatory response (Morris et al., 2020). Similarly, RPE-secreted exosomes have been reported to contain angiogenic factors promoting CNV (Hajrasouliha et al., 2013).

RPE-secreted EVs could also impact RPE cell health and function directly and contribute to AMD pathophysiology via autocrine signaling. For example, RPE derived exosomes have been reported to induce epithelial-to-mesenchymal transition (Zhang et al., 2020). Furthermore, RPE-derived exosomes are enriched in AMD-associated proteins like CD46 and CD59 that play an important role in local complement regulation within the RPE monolayer. In fact, exposure to cigarette smoke, the biggest modifiable risk factor for AMD (Velilla et al., 2016; Dalvi et al., 2019), resulted in increased levels of AMD-associated proteins, including several drusen resident proteins in exosomes secreted by the RPE (Wang et al., 2009b; Flores-Bellver et al., 2021). In demonstrating drusen-associated proteins are secreted in exosomes from RPE cells, Flores-Bellver et al., 2021., rationalized interrogating a causal role of RPE-secreted exosomes in promoting AMD pathophysiology. Consistently, a recent study (Kurzawa-Akanbi et al., 2022) showed that supplementation of control iPSC-RPE cultures with AMD iPSC-RPE-secreted apical EVs is independently sufficient to cause AMD-associated features such as stress vacuoles, cytoskeletal destabilization and abnormal morphology of the nucleus in control iPSC-RPE cells. Furthermore, this study documented a vast difference in the transcriptomic, proteomic and lipidomic profile of EVs released by control iPSC-RPE and AMD iPSC-RPE cells (Kurzawa-Akanbi et al., 2022).

Oxidative stress has been directly shown to both increase the exosome secretion by the RPE cells (Atienzar-Aroca et al., 2016) and affect the composition of RPE-secreted exosomes (Biasutto et al., 2013); thereby potentially contributing to drusen formation and aberrant angiogenesis in AMD (Kannan et al., 2016; Tong et al., 2016; Atienzar-Aroca et al., 2018; Shah et al., 2018; Maisto et al., 2019; Ke et al., 2020). Accordingly, RPE-secreted exosomes have been reported to contain several angiogenic proteins (e.g., MMP9 and VEGFA) (Fukushima et al., 2020) and various other phosphoproteins (e.g., AKT1 and MAPK14) (Biasutto et al., 2013). Furthermore, it has been shown that oxidative stress contributes to autophagy induction, immune dysregulation, inflammation, and angiogenesis in AMD-relevant cell type(s), endothelial cells, photoreceptors, and muller glial cells by inducing changes in the phosphorylation status of several proteins, including proteins involved in cell metabolism like AMPK α1 (S485) and ACACA (S79) inside the exosomes (Biasutto et al., 2013; Atienzar-Aroca et al., 2016; Tong et al., 2016; Todorova et al., 2017; Li et al., 2019; Fukushima et al., 2020; Ke et al., 2020).

Oxidative stress also has the potential to alter EV shedding as lipid peroxidation affects plasma membrane fluidity and consequently lipid-raft-like domains that are enriched in cholesterol and sphingolipids (Totsuka et al., 2019). In support of this notion, lipidomic studies of EVs isolated from several cells types (e.g., PC-3 cells, B-lymphocytes) showed 2- to 3-fold enrichment of cholesterol, sphingomyelin, glycosphingolipids, and phosphatidylserine in the exosomes compared to cells (Skotland et al., 2017).

Oxidative stress has also been reported to cause mitochondrial dysfunction and aberrant dysregulation of mitochondrial miRNA (Brown et al., 2019). Notably, RPE cells treated with rotenone, a potent inhibitor of complex I of the mitochondrial respiratory chain, showed an increased number of EVs containing mitochondrial miRNA miR-494-3P, which regulates several proteins involved in mitochondrial biogenesis, such as TFAM and SIRT1 (Lin et al., 2018; Ahn et al., 2021)

EVs have also been shown to modulate inflammation in the retina. Consistently, cytokines (Gao et al., 2019; Jung et al., 2020), inflammasome (Liu et al., 2016; Zhang W et al., 2019), and complement system proteins (Datta et al., 2017) that are present in the EVs and have been postulated to directly contribute to AMD pathogenesis (Ke et al., 2020; Otsuki et al., 2021). For example, RPE-released EVs are enriched in proteins that regulate immune response, inflammation, and the complement system (Kang et al., 2014; Klingeborn et al., 2017; Flores-Bellver et al., 2021). Furthermore, injecting exosomes secreted by ARPE-19 cells under oxidative stress in the vitreous led to an inflammatory response and caspase-9-mediated apoptosis in the rat retina (Ke et al., 2020). Similarly, consistent with a plausible role of RPE-released EVs in promoting sub-retinal inflammation in AMD, CD63^+^ exosomes secreted by murine primary RPE cells led to increased secretion of several proinflammatory cytokines including MCP1, IL6, VEGFA, and TNF TNFAIP1in a co-culture model system consisting of primary murine RPE cells and a macrophage cell line RAW 264.7 (Otsuki et al., 2021).

The increased shedding and quantity of EVs during inflammatory conditions, along with appearance of complement products, suggests a strong association between the complement system and EVs (Yamamoto et al., 2015). Also, many complement proteins such as C1QA, C3, C4B, C5, CD46, and DAF have been reported in EVs (Winston et al., 2019); reviewed extensively in (Karasu et al., 2018). With regard to the retina, specific complement proteins have been identified in RPE-secreted EVs, including C3, C4B, CFB, CFI, C1R, C1S, and C1QTNF (Klingeborn et al., 2017; Flores-Bellver et al., 2021). Furthermore, the complement proteins present in RPE-released EVs were shown to increase in the presence of AMD-relevant stressors (e.g., C3, C4A, C4B) and accumulate in the drusen or ECM beneath the RPE monolayer (Wang et al., 2009a; 2009b; Klingeborn et al., 2017; Flores-Bellver et al., 2021). It is plausible that the accumulation of these EVs plays an important role in the recruitment of macrophages and other immune cells and subsequently amplification of complement activation and inflammation. It is also possible that due to loss of choriocapillaris, there is increased build-up of the circulating EVs and consequently, deposition of membrane attack complex (C5b-9) leading to the release of mitogenic factors and cytokines that cause pathological cell proliferation in AMD (Huang et al., 2018). Another possibility is that risk associated genetic variants for AMD, like *CFH Y402H* and *C3 R102G,* promote leukocyte attack on C3-coated exosomes leading to excess release of exosomal content that then plays a causal role in drusen formation (Wang et al., 2009a; 2009b).

Inflammasomes are multimeric protein complexes which on activation release cytokines and consequently mediate local and systemic inflammation in several diseases including AMD (Barker et al., 2011; Celkova et al., 2015; Zhang W et al., 2019; Chan and Schroder 2020). For instance, canonical inflammasomes like NLRP3 depend on caspase-1-dependent processing and secretion of pro-inflammatory cytokines like IL1B and IL18 (Barker et al., 2011). Both IL1B and IL18 precursors that are directly linked to inflammation in AMD (Chan and Schroder 2020) are present in EVs (Jung et al., 2020; Gao Q et al., 2021). Furthermore, a direct association between EV secretion and inflammasome-mediated inflammation has been reported (Celkova et al., 2015), although the mechanism of “how” the inflammasome activity regulates secretion of EVs and modulates EV cargo is currently not known. One possible hypothesis supported by published literature is that oxidative stress activates the NLRP3 inflammasome pathway in RPE and thereby leads to increased autophagy and subsequently, enhanced secretion of EVs containing ECM- and drusen-associated proteins (Sreekumar et al., 2010; Biasutto et al., 2013; Hajrasouliha et al., 2013; Knickelbein et al., 2016; Atienzar-Aroca et al., 2018; Maisto et al., 2019; Zhang W et al., 2019; Fukushima et al., 2020; Ke et al., 2020; Nicholson et al., 2020; Wooff et al., 2020; Ahn et al., 2021; Flores-Bellver et al., 2021; Maldonado et al., 2021; Martins et al., 2021).

Overall, both *in vivo* models and cell culture studies (primarily utilizing RPE cells) support a role of EVs in modulating AMD pathophysiology via autocrine and paracrine signaling.

#### 2.3.2 DR

DR is a microvascular complication of diabetes that leads to vision loss. Almost all type I diabetes patients develop DR and two-thirds of patients with type 2 diabetes have some form of DR (Fong et al., 2004). Abnormal glucose levels in diabetes can affect different retinal cells and can cause abnormal angiogenesis, blood-retinal barrier (BRB) breakdown, gliosis, inflammation, and ultimately fibrosis (Fong et al., 2004; Duh et al., 2017; Yang et al., 2022). Furthermore, EV-mediated crosstalk between various retinal cell types (e.g., retinal astroglia cells, RPE, pericytes, endothelial cells, retinal neurons, astrocytes, etc.) has been shown to influence levels of inflammatory/angiogenic mediators in diabetes and contribute to DR development (Huang 2020). For example, exosome-mediated transfer of cPWWP2A from pericytes to endothelial cells led to retinal vascular dysfunction in a streptozotocin-induced diabetic mouse model (Liu C et al., 2019). Similarly, another circular RNA, circEhmt1, was upregulated in pericyte-derived exosomes and protected endothelial cells from high glucose mediated stress via the NLRP3 pathway (Ye et al., 2021). Likewise, both *in vitro* and *in vivo* experiments validated that aberrant expression of miR-9-3p in Müller glia-derived exosomes aggravate vascular dysfunction under high glucose conditions (Liu Y et al., 2022).

EVs secreted from specific retinal cell type(s) have also been shown to be enriched in anti-angiogenic proteins. For example, exosomes isolated from retinal astroglia cells have been shown to include anti-angiogenic molecules like COL18A1 and SERPINF1. Similarly, astrocyte-derived EVs have also been reported to contain antiangiogenic proteins (COL18A1, SERPINF1, and TIMP1) (Hajrasouliha et al., 2013). Furthermore, it has been shown that pathologically-relevant cytokine(s) influence the content of astrocyte-derived EVs (Dickens et al., 2017). Specifically, upregulation of IL1B and IL10 in the vitreous of PDR patients (Mao and Yan 2014) differentially influences the composition of astrocyte-derived EVs (Chaudhuri et al., 2018). Astrocytes exposed to IL10 secrete EVs that promote neuronal survival due to the presence of neuroprotective molecules such as FGF14, PGRMC1, CS, miR-125a-5p, and miR-16-5p (Chaudhuri et al., 2018; Datta Chaudhuri et al., 2020). In contrast, astrocytes treated with IL1B contain pro-inflammatory modulators, including C3, PTMA, LOX, and micro RNAs (let-7f, miR-24, and miR-100) (Datta Chaudhuri et al., 2020). Notably, exposure to cigarette smoke, a known risk factor for DR (Moss et al., 1991), led to increased levels of pro-angiogenic factors (e.g., VEGF, MMP9, TIMP1, IGFBP3) in another DR relevant cell type, the RPE (Hajrasouliha et al., 2013; Fukushima et al., 2020).

It has also been postulated that exosomes from the plasma cross the blood-retinal barrier and activate the classical complement pathway, which in turn contributes to DR development (Huang et al., 2018; Huang et al., 2020). Comparative study of plasma collected from control and streptozotocin-induced diabetic mice showed a significant increase in the concentration of exosomes in plasma of diabetic mice at the 3-month timepoint (Huang et al., 2018). Furthermore, proteomic analysis has shown the presence of pro-inflammatory and pro-angiogenic proteins in EVs isolated from both VH and plasma of DR patients (Safwat et al., 2018; Yang et al., 2022). Consistently, mouse endothelial cells exposed to exosomes isolated from either the serum of diabetic mice or diabetic patients displayed impaired vascular function (Zhang H et al., 2018). Similarly, miR-9-3p, a microRNA in exosomes isolated from VH of patients with proliferative DR promoted cell proliferation, migration, and tube formation in cultured human retinal endothelial cells (Liu Y et al., 2022). TNFAIP8, which is upregulated in both plasma EVs and vitreous of DR patients, has also been proposed to be an important mediator of aberrant angiogenesis in DR (Xiao et al., 2021).

Exosomes have also been implicated to play a role in DR via the modulation of endocrine signaling (Kamalden et al., 2017). Specifically, using high fat diet and pancreatic β-cell-specific miR-15a^/-^ mice, it was shown that miR-15a-containing exosomes released from pancreatic β-cells cross the inner blood-retina barrier and activate Müller cell apoptosis through the AKT-3 pathway (Kamalden et al., 2017). Exosomes could also initiate/promote DR pathogenesis by influencing development of insulin resistance/diabetes, as high fat diet has been reported to alter the lipid composition of EVs (Kumar et al., 2021). Corroborating this postulation, lean mice became insulin resistant after being administered exosomes isolated from feces of either high fat diet fed obese mice or patients with type II diabetes (Kumar et al., 2021).

Overall, a multitude of clinical, animal model, and *in vitro* studies provide support for a role of both systemic and retina-associated EV alteration in DR pathobiology.

#### 2.3.3 Other retinal diseases

Glaucoma is associated with optic nerve damage, retinal ganglion cell (RGC) death, and increased intraocular pressure. Mechanistically, the blockage of aqueous humor drainage leads to increased intraocular pressure, causing optic nerve damage in glaucoma. In direct consequence to glaucoma pathogenesis, exosomes released from non-pigmented ciliary epithelial cells regulated the pore size of trabecular meshwork cells and promoted aqueous humor drainage flow (Tabak et al., 2021). Consistent with the aforementioned therapeutic effect, proteome and miRNA analysis of non-pigmented ciliary epithelial cell-released exosomes revealed 584 miRNAs and 182 proteins involved in the modulation of trabecular meshwork cellular processes, including the WNT/β-catenin signaling pathway, cell adhesion, and ECM deposition (Lerner et al., 2017; Lerner et al., 2020). There is also evidence of cytokines, drugs, and oxidative stress affecting the ocular drainage system by impacting exosome release (Hardy et al., 2005; Hoffman et al., 2009; Lerner et al., 2017; Lerner et al. 2020; Lerner et al., 2020; Tabak et al., 2021; Takahashi et al., 2021). For example, in the presence of oxidative stress induced by AAPH (2,2′-Azobis (2-methylpropionamidine) dihydrochloride), non-pigmented ciliary epithelial cell exosomes have a protective effect on trabecular meshwork cells (Lerner et al., 2020). Similarly, the treatment of trabecular meshwork cells with dexamethasone increased mycolin-mediated exosome release and reduced the amount of fibronectin bound per exosome (Hardy et al., 2005; Hoffman et al., 2009; Dismuke et al., 2016). It has also been shown that TGFß2-treated trabecular meshwork cells show altered EV miRNA expression that promotes glaucoma pathogenesis (Zhang and Wang 2021).

Microglia-mediated neuroinflammation is one of the early events in glaucoma pathology. Notably, murine microglial cells increased exosome secretion when exposed to elevated hydrostatic pressure that mimics the intraocular pressure (Aires et al., 2020). Furthermore, consistent with EVs released from microglial cells under elevated pressure contributing to glaucoma pathology, microglia-released exosomes acting in an autocrine manner increased inflammation, phagocytosis, and migration (Hazrati et al., 2022). Also, demonstrating the ability of microglia-secreted EVs to influence retinal pathology in other inflammatory diseases, treatment of murine microglial cells with lipopolysaccharide (LPS)/IFN- γ upregulated expression of several anti-inflammatory cytokine mRNAs in the released EVs, that then ameliorated the disease phenotype when injected in a mouse glioma model (Grimaldi et al., 2019).

Retinopathy of prematurity (ROP) is a retinovascular disease that occurs in premature infants where retinal vasculature abnormally migrates into the vitreous instead of the normal growth towards the ora serrata. Decreased VEGF and TGFß expression and smaller avascular areas with fewer neovascular tufts were observed when microglia-derived exosomes were injected into the vitreous of a mouse model of oxygen-induced retinopathy (Xu et al., 2019). This supports a protective role for microglia-released exosomes in ROP.

Overall, both *in vitro* and *in vivo* studies support a role of EVs in multiple retinal diseases.

## 3 EVs as potential biomarkers for retinal degenerative diseases

Altered composition of EVs under pathological conditions, their varied molecular cargo, and their accessibility from biofluids make them ideally suited as a diagnostic and prognostic biomarker. It is also noteworthy that mutations present in parental DNA and the proteome are consistent with the DNA and proteins of EVs in different disease models (Degli Esposti et al., 2021). In contrast, the RNA profiles of EVs differed substantially from those of their cells of origin (Veziroglu and Mias 2020), suggesting that some RNA species are selectively incorporated into EVs. Thus, identifying disease-associated alterations in patient derived EVs would be valuable for biomarker development. Similarly, understanding how diseased state impacts EV bio-distribution (Section 3.1) is important to consider for the utilization of EVs as a biomarker.

### 3.1 Bio-distribution of EVs

Recent studies have shown that it is possible to trace the origin of EVs in biofluids based on the composition of EVs and their interaction with the recipient cells (Larssen et al., 2017; Zhang Y et al., 2019; Hamzah et al., 2021; Garcia-Martin et al., 2022). For example, using quantitative proteomics, the cellular origin of serum exosomes/sEVs were identified (Garcia-Martin et al., 2022). Similarly, using multiplex proximity extension assays followed by the proteomic profiling helped identify the cellular origin of EVs in seminal plasma and human milk (Larssen et al., 2017). Furthermore, *in vivo* properties of EVs, such as tissue bio-distribution, blood level, urine clearance, and time taken to reach its target site, were studied in nude mice using a multimodal imaging reporter (Lai et al., 2014). In the aforementioned study, EVs isolated from HEK293T cells expressing Gaussia luciferase reporter injected via tail vein preferentially localized in the spleen and the liver of the mice within 30 min. Furthermore, EV post-systemic injections were cleared via hepatic and renal routes within 6 h of the injection (Lai et al., 2014). Ultimately, ability to trace the source of the cellular origin of EVs in biofluids (e.g., urine, serum, plasma, saliva, tear, vitreous and aqueous humor) makes them an ideal candidate for biomarker-based studies, and also potentially a suitable platform for investigating the system level physiology and pathophysiology of specific diseases (Palviainen et al., 2020; Liang et al., 2021).

### 3.2 EVs as a biomarker in retinal disease

Proteomic alterations in ocular biofluids like VH have been documented in several retinal diseases as described above (*se*e Section 2.1). Thus, it is plausible that exosomes isolated from the VH and other ocular biofluids could assist in early diagnosis of specific retinal degenerative diseases and serve as an outcome measure for evaluating disease progression and therapeutic intervention. Furthermore, given the involvement of systemic factors in the development and progression of specific retinal degenerative diseases, like AMD, it is plausible that circulating EVs in human plasma and serum could serve as biomarkers for these diseases. Consistently, exosomal miRNAs (miR-19a, miR-126, miR-410) isolated from the serum of AMD patients have been proposed as biomarkers (Li et al., 2014; ElShelmani et al., 2021). Similarly, proteomics of exosomes isolated from aqueous humor of AMD patients showed increased abundance of proteins involved in *i)* the autophagy-lysosomal pathway (e.g., Cathepsin D, CTSD, complement proteins) and *ii)* epithelial-mesenchymal transition (e.g., Actin Alpha 2, Smooth Muscle, ACTA2, Myosin IF, MYO1F) (Kang et al., 2014). Consistent with these exosomal proteins in the aqueous humor serving as a suitable biomarker(s) for disease progression and therapeutic intervention, levels of these proteins were decreased in the exosomes isolated from AMD patients treated with ranibizumab (Kang et al., 2014). Similarly in DR, TNFAIP8 was upregulated in both plasma and vitreous EVs, and therefore could potentially serve as a biomarker for DR (Xiao et al., 2021).

Although the use of exosomes as a biomarker is not yet utilized clinically for retinal degenerative diseases, several clinical trials for other diseases are currently evaluating exosomes as a diagnostic tool (e.g., NCT05286684, NCT04529915). We anticipate that an enhanced understanding of EV composition in healthy and diseased retina, and the tracing of the cellular origin of exosomes in ocular biofluids (VH, tear, aqueous humor) and plasma will be instrumental for the identification of novel biomarkers for several retinal diseases.

## 4 EV-based therapeutics for retinal diseases

Specific characteristics of EVs, including the nano-scale size, bioavailability, biocompatibility, and ability to modulate crosstalk between cells, makes them ideally suited for therapeutic intervention. This is especially relevant to retinal degenerative diseases that often display multi-tissue pathology and involve crosstalk between several retinal cell type(s). Furthermore, EVs can themselves be therapeutic or serve as delivery vehicles for encapsulated therapeutic molecules (drugs, peptides, small molecules) and notably both of these approaches have been utilized for several eye diseases including specific retinal disease (Table 3). (Aboul Naga et al., 2015; Moisseiev et al., 2017; Zhu et al., 2017; Zhou et al., 2018; Xu et al., 2019; Bian et al., 2020; Kang et al., 2020; Dong et al., 2021; Gu et al., 2021; Tian et al., 2021; Wang T et al., 2021; Wang W et al., 2021; Wang Y et al., 2021; Zhou et al., 2021; Liang et al., 2022; Lin et al., 2022; Liu Y et al., 2022; Pollalis et al., 2022; Ren et al., 2022; Zhou et al., 2022)

**TABLE 3:** Published studies on use of EVs a therapeutic delivery vehicle and/or therapeutic molecule for targeting retinal diseases.

Type of EVs	Source of EVs (Cellular or animal model)	Delivery molecules	Recipient	Disease model	References
Exosomes	RGC	Peptides (pituitary adenylate cyclase-activating polypeptide 38)	Rat	Traumatic optic neuropathy	*(Wang T et al. 2021)*
Exosomes	Mouse retina tissue	Peptide (RGD)	Mouse	Laser-induced CNV	*(Pollalis et al. 2022)*
Exosomes	HEK293T	Retinoschisin1 (RS1)	Mouse	Not applicable	*(Wang W et al. 2021)*
Exosomes	HUVEC	Anti-Angiogenic peptide-KV11	Mouse	Oxygen induced retinopathy	*(Dong et al. 2021)*
Exosomes	Mouse retinal progenitor cells	Cre^+^ Exosomes	Mouse	Not applicable	*(Zhou et al. 2018)*
Exosomes	BMSC	Growth factors and angiogenic proteins	Mouse	Oxygen-induced retinopathy	*(Moisseiev et al. 2017)*
Exosomes	HEK293T	miR-199a-3p-chimeric protein FLAG peptide, TAT peptide	Mouse	Not Applicable	*(Zhu et al. 2017)*
Exosomes	Porcine RPE and ARPE-19	Bevacizumab	ARPE-19	Laser-induced CNV	*(Aboul Naga et al. 2015)*
Exosomes	Photoreceptor cells (661w)	AMPK-activated protein kinases	Mouse	Streptozotocin induced diabetes	*(Ren et al. 2022)*
Exosomes	Microglial cells BV2	miR24-3p	Mouse	Oxygen-induced retinopathy	*(Xu et al. 2019)*
Exosomes	ARPE-19	exosomes	Mouse	MNU-induced retinal degeneration	*(Wang Y et al. 2021)*
Exosomes	Neural progenitor cells	miRNAs	Rat	Retinal degeneration	*(Bian et al. 2020)*
Exosomes	BMSC	miR-133b-3p	Mouse	Diabetic retinopathy	*(Liang et al. 2022)*
Exosomes	Mesenchymal stromal cells	miR-204	Mouse/patient	Dry eye	*(Zhou et al. 2022)*
Exosomes	T regulatory cells	Anti-VEGF antibody	Mouse	Laser-induced CNV	*(Tian et al. 2021)*
EVs	Rat adipose derived MSC	miR-192	Mouse	Streptozotocin induced diabetes	*(Gu et al. 2021)*
Exosomes	HEK293T	Aptamer S58	Rat	Glaucoma	*(Lin et al. 2022)*
Exosomes	Mouse muller glial cells	miR-9-3p	Mouse	Diabetic retinopathy	*(Liu Y et al. 2022)*
Exosomes	Mouse B-regulatory cells	IL35	Mouse	Encephalomyelitis/uveitis	*(Kang et al. 2020)*

To date, two different modes of encapsulating molecules, active and passive, have been used with EVs. Active encapsulation includes sonication of drugs or molecules in the cells. Various drugs, such as doxorubicin and paclitaxel, have been loaded into the EVs using approaches where incorporation of drugs in the outer membrane of the exosome results in an initial burst phase of release followed by a second phase of slow release. (Haney et al., 2015; Schindler et al., 2019). Other methods of active encapsulation include extrusion, where the exosome membrane is disrupted for drug incorporation. In contrast to active encapsulation approaches, passive encapsulation involves incubation of the therapeutic molecules with the EVs and results in concentration-dependent incorporation of drugs/molecules into the EVs. Note that with this approach, the hydrophobicity of the molecules being encapsulated determines the loading capacity. For example, passive encapsulation for drug delivery has been used experimentally where curcumin and enzyme catalase were encapsulated in exosomes and delivered intranasally to a mouse model of Parkinson disease (Haney et al., 2015). Another method for obtaining drug-loaded exosomes is isolating the exosome from the drug-treated cells. Consistent with the efficacy of this approach, exosomes isolated from paclitaxel-treated mesenchymal stromal cells showed strong anti-proliferative effect in three different cancer cell lines (acute lymphoblastic leukemia, glioblastoma, and prostate carcinoma) (Pessina et al., 2011).

Although the use of exosomes as a drug delivery vehicle in the retina is still in preliminary stages, the anatomic position of the retina is conducive for testing EVs as carrier molecules for various therapeutic agents (e.g., peptides, nanoparticles, liposomes). Remarkably, RGC-derived exosomes loaded with pituitary adenylate cyclase-activating polypeptide 38 (PACAP38) enhanced the RGC survival rate, increased the thickness of the retinal nerve fiber layer, promoted axon regeneration, and ultimately improved optic nerve function after injury (Wang T et al., 2021). Similarly, exosomes from human umbilical vascular endothelial cells (HUVECs) loaded with antiangiogenic peptides (KV-11) showed anti-angiogenic activity in a mouse model of oxygen-induced retinopathy (Dong et al., 2021).

Conventional adeno-associated virus (AAV) vectors have only limited transduction efficiency after intravitreal injection with the inner limiting membranes acting as a major barrier. Exosome-associated AAV2 vectors outperformed conventional AAV2 in retinal transduction after intravitreal injection and were able to transduce a high number of bipolar cells, and also some photoreceptors (Wassmer et al., 2017). Similarly, exosome-associated AAV retinoschisin 1 (*RS*1) vector improved the transduction efficiency of RS1 (Wang W et al., 2021).

EVs themselves have been routinely utilized as a therapeutic molecule in both *in vitro* cell culture platforms as well as pre-clinical animal models of several retinal diseases. In a rabbit model of DR, mesenchymal stem cell (MSC)-derived exosomes induced retinal repair through miR-22 (Safwat et al., 2018). Similarly, sub-retinal delivery of ARPE-19-derived exosomes alleviated structural damage and visual function impairments in an N -methyl- N -nitrosourea (MNU)-induced mouse model of retinal degeneration (Wang Y et al., 2021). Likewise, direct administration of mouse neural progenitor cell-derived exosomes (mNPC-exosome) delayed photoreceptor degeneration, preserved visual function, and prevented thinning of the outer nuclear layer in the Royal College of Surgeons (RCS) rats (Bian et al., 2020). Mechanistically, this was due to the internalization of mNPC-exosomes by the retinal microglia, which suppressed their activation. Recently, bone marrow mesenchymal stem cell (BMSC)-derived exosomal miR-133b-3p suppressed angiogenesis and oxidative stress in a mouse model of DR (Liang et al., 2022). Furthermore, BMSC-derived exosomes co-cultured with high glucose-treated Muller glial cells inhibited oxidative stress, inflammation, apoptosis, and promoted cell proliferation in a DR model (Li W et al., 2021).

EVs isolated from MSCs derived from human embryonic stem cells also showed neuroprotective and axiogenic effects in protecting retinal function in an optic nerve crush mouse model (Seyedrazizadeh et al., 2020). Specifically treatment with MSC-derived EVs downregulated cis-p tau protein, prevented retinal nerve fiber degeneration, increased GAP43^+^ axon counts, and promoted cognitive visual behavior in this study (Seyedrazizadeh et al., 2020). Exosomes derived from MSCs and other cells have also been reported to have a protective effect against oxidative stress in trabecular meshwork cells in glaucoma (Lerner et al., 2020; Li Y-C et al., 2021). Similarly, B-regulatory cell released exosomes that contain IL35 have been reported to suppress neuroinflammation in an autoimmune uveitis model (Kang et al., 2020). Notably, exosomes derived from T-regulatory cells cross-linked with anti-VEGF antibody via a matrix metalloproteinases cleavable peptide linker markedly suppressed ocular neovascularization in mouse and non-human primate models of CNV (Tian et al., 2021).

EVs have also been reported to play a central role in paracrine effects of transplanted stem cells (Bian et al., 2020). For instance, RGC survival rates were increased significantly in a rat optic nerve crush model when treated with bone marrow derived stem cells (Da Silva-Junior et al., 2021). Consistent with a protective role of exosomes in RGC survival in this model, the therapeutic effects of exosomes were diminished after knockdown of Argonaute-2, a key miRNA effector molecule present in the exosomes (Mead and Tomarev 2017).

Altogether, EVs, apart from acting as drug delivery vehicles, can serve as therapeutic molecules themselves and be utilized for autocrine, paracrine, or endocrine targeting. However, because EVs contain various proteins, RNA, DNA, lipids, etc. as cargo, they can have off-target effects on both the intended recipient and other cell type(s). Thus, it will be important that before using EVs for therapeutic purposes targeting a tissue like the retina that biological characteristics are investigated in detail, and both beneficial and negative consequences are verified in pre-clinical models.

## 5 Conclusion and future directions

**FIGURE 1:**
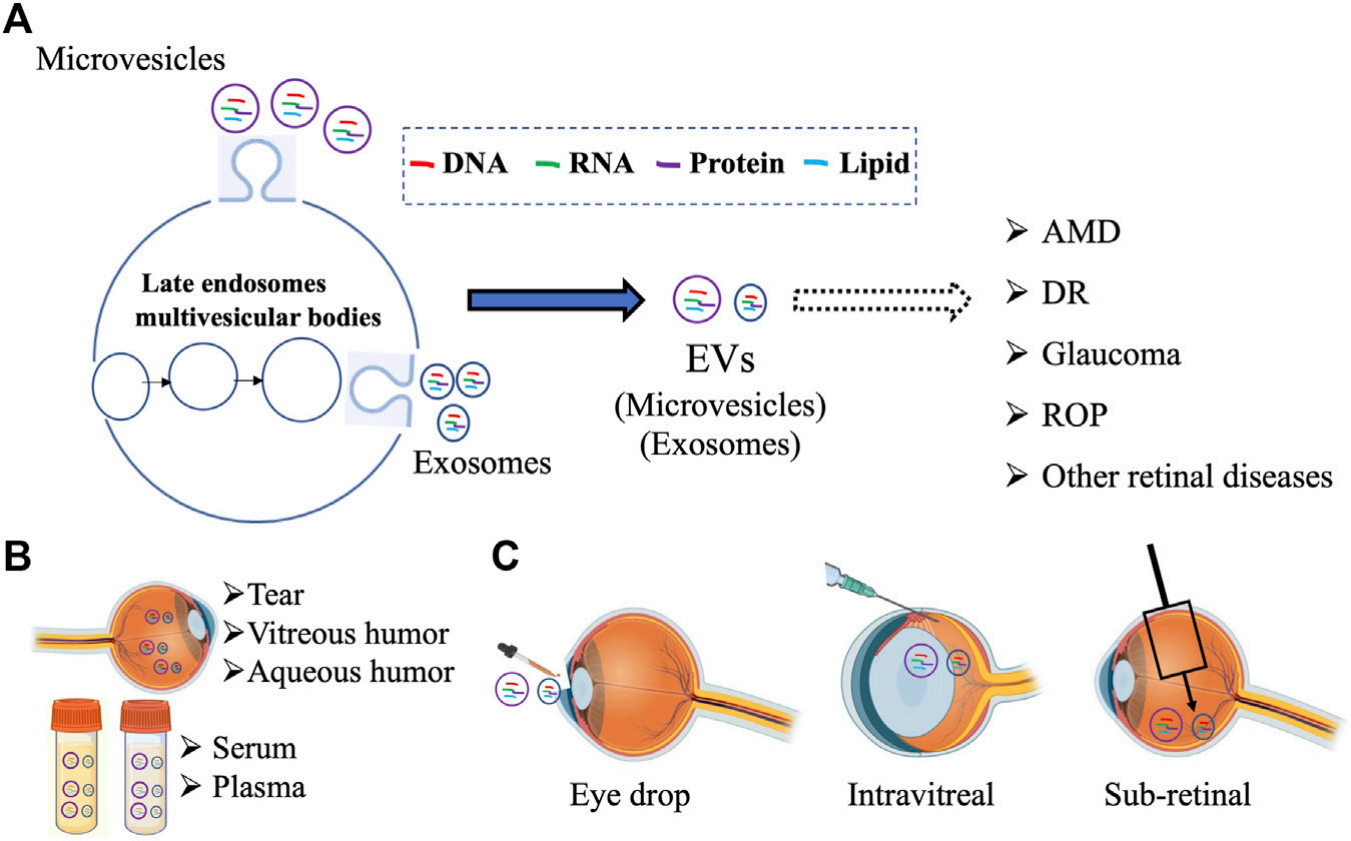
Schematic summarizing the multiple avenues of research involving EVs in the retina including **(A)** examining the contribution different subpopulation of EVs (microvesicles and exosomes) in retinal pathophysiology, **(B)** developing EV based biomarker for ocular diseases from different biofluids, and **(C)** targeting retinal diseases with EV-mediated therapies. Figure was prepared using bioRender®.

EVs are multifunctional molecules that can affect cell and tissue homeostasis in autocrine, endocrine, and paracrine manners. Although the research on the role of EVs in retinal (patho)physiology is still nascent, several recent studies have utilized both *in vivo* and *in vitro* approaches to investigate “how” EVs contribute to retinal homeostasis. Notably, the presence of EVs has been documented in several ocular biofluids and compositional changes in VH and aqueous humor have been reported for specific retinal diseases (Section 2.1). Furthermore, utilizing both cell culture systems and relevant animal models, several studies have now provided preliminary evidence that support a role of EVs, particularly exosomes, in several retinal diseases (Figure 1; Sections 2.2, 2.3). Multiple studies have also investigated the utility of EVs as a biomarker and potential therapeutic agent and drug delivery vehicle (Figure 1; Sections 3, 4).

**FIGURE 2:**
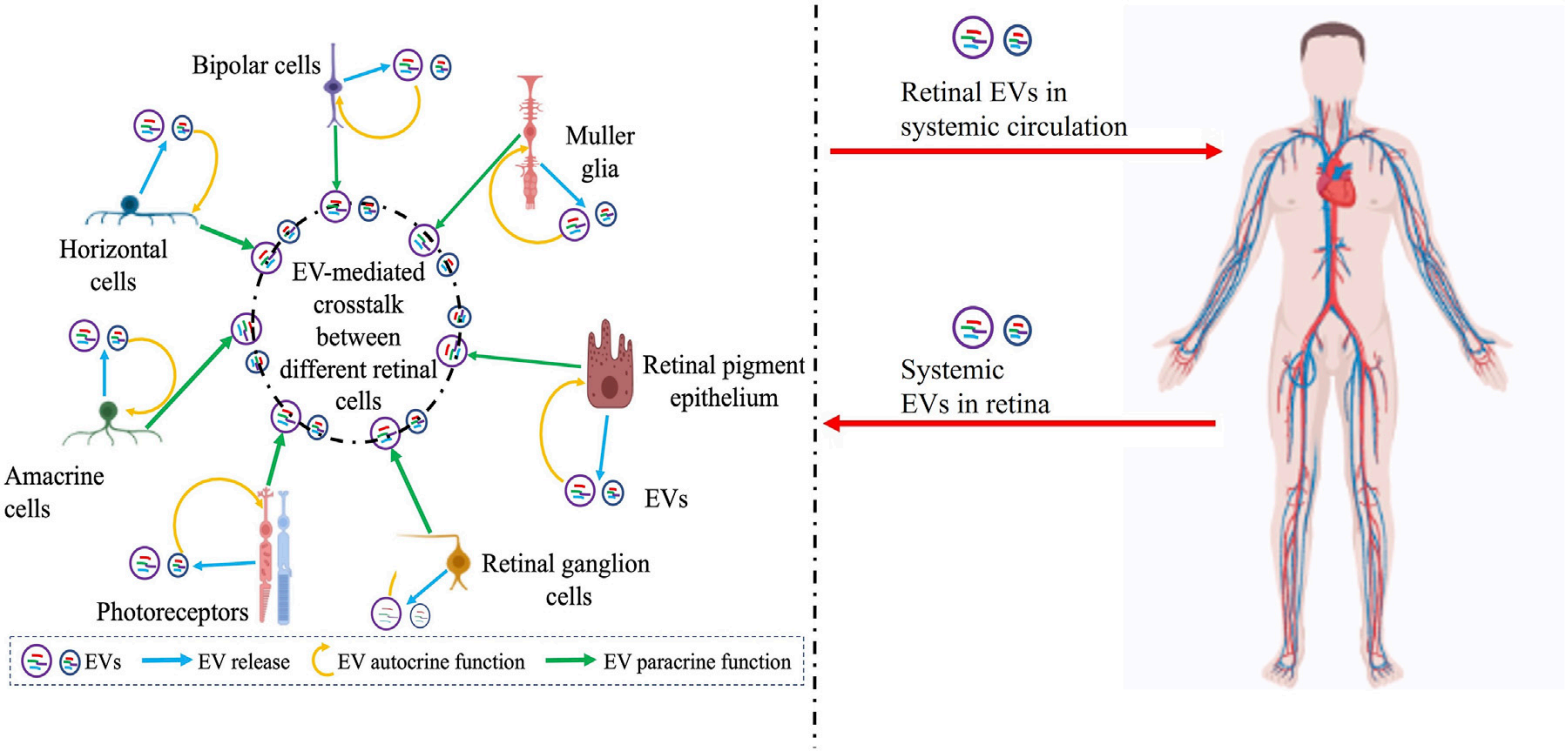
Schematic showing the complex interplay of EVs in the retina with several confounding factors including potential systemic contribution of EVs that need to be further articulated to precisely understand the role of EVs in retinal (patho)physiology, develop rational biomarkers, and target retinal diseases with EV-mediated therapies. Potential EV-mediated autocrine paracrine and signaling between different retinal cell types is illustrated on the left and the possibility of retinal EVs in systemic circulation or EVs from systemic circulation within the retina is indicated on the right. Figure was prepared using bioRender®.

Despite these advances, our understanding of the precise role of specific types of EVs (e.g., microvesicles *versus* exosomes) and cell-specific EV cargo in retinal homeostasis is limited (Figures 1, 2). For example, we do not know “whether” or “how” alterations in EVs at the systemic (e.g., plasma) *versus* local (e.g., retina) level are pathologically relevant in specific retinal diseases (Figure 2). Even within the retina, the complexity of distinct type(s) of EVs (Table 1) being released from multiple retinal cell type(s) and the potential of EVs secreted from specific cell type(s) to modulate retinal function via both autocrine and paracrine effects will be challenging to decipher (Figure 2). The consideration of EV bio-distribution and its regulation via multiple factors, including nearby and distant cells, and the inability to definitively determine cause-effect relationships *in vivo* will be major impediments for precisely understanding the role of EVs in retinal homeostasis. The challenges of identifying the cellular source of EV origin and the targets of EVs secreted from a specific cell type (Figure 2) not only hampers our ability to study the role of EVs in retinal (patho)physiology, but also poses challenges for the use of EVs in therapeutic approaches, including as delivery vehicles or medicinal molecules by themselves. This is particularly important from the perspective that distinct EVs contain a diverse molecular cargo (Table 1) and thus, there is a potential of deleterious off-target effects on both the intended recipient and other cell type(s). However, it is expected that future studies focused on identifying different sub-populations of EVs in the retina with cell-type specificity in normal *versus* diseased condition(s) will help address these big gaps in knowledge. Similarly, understanding the consequence of cell-specific EV cargo (e.g., DNA, RNA, protein, lipid) and understanding the mechanism behind the packaging of this cargo for retinal homeostasis will be an important consideration for future studies.

The identification and characterization of cell specific EVs in body fluids like plasma, serum, VH, and aqueous humor in healthy subjects *versus* patients with retinal degenerative diseases will also be instrumental in development of biomarkers (Figure 1). iPSC technology will similarly provide a unique platform suitable to study EV biology in isolated retina cell-type(s), retina co-culture model(s), and retinal organoids that are not accessible in *in vivo* bio-fluids like VH. This would be especially relevant in understanding the role of EV-mediated intercellular communication to modulate the multi-tissue pathology of complex retinal degenerative diseases like AMD. For example, we recently utilized the “modularity” of the iPSC model system that allows investigation of a single cell type in disease pathophysiology to show that dysfunctional RPE in the retina is sufficient for the development of key pathological features of macular degeneration (Galloway et al., 2017; Manian et al., 2021) Notably, utilizing the 3D RPE-choriocapillaris model, we showed that RPE-secreted factors independently contributed to CNV-like pathology, thus providing a suitable model system to interrogate the causal *versus* protective role of RPE-secreted exosomes in disease pathology development. An additional advantage of the iPSC-derived model(s) of the retina platform is the ability to test EV-based therapeutics in the patient’s own cells.

Altogether, we are embarking on a new EV revolution with several unanswered questions that has the potential to 1) revolutionize our understanding of retinal (patho)physiology and 2) provide EV-based biomarkers and therapies for specific retinal diseases.
